# Segmentation and modeling of large-scale microvascular networks: a survey

**DOI:** 10.3389/fbinf.2025.1645520

**Published:** 2025-10-31

**Authors:** Helya Goharbavang, Artem T. Ashitkov, Athira Pillai, Joshua D. Wythe, Guoning Chen, David Mayerich

**Affiliations:** 1 Department of Electrical Engineering, University of Houston, Houston, TX, United States; 2 Department of Cell and Developmental Biology, University of Virginia School of Medicine, Charlottesville, VA, United States; 3 Department of Computer Science, University of Houston, Houston, TX, United States

**Keywords:** segmentation, skeletonization, vascular, microvascular, network

## Abstract

Recent advances in three-dimensional microscopy enable imaging of whole-organ microvascular networks in small animals. Since microvasculature plays a crucial role in tissue development and function, its structure may provide diagnostic biomarkers and insight into disease progression. However, the microscopy community currently lacks benchmarks for scalable algorithms to measure these potential biomarkers. While many algorithms exist for segmenting vessel-like structures and extracting their surface features and connectivity, they have not been thoroughly evaluated on modern gigavoxel-scale images. In this paper, we propose a comprehensive yet compact survey of available algorithms. We focus on essential features for microvascular analysis, including extracting vessel surfaces and the network’s associated connectivity. We select a series of algorithms based on popularity and availability and provide a thorough quantitative analysis of their performance on datasets acquired using light sheet fluorescence microscopy (LSFM), knife-edge scanning microscopy (KESM), and X-ray microtomography (µ-CT).

## 1 Introduction

Microvasculature plays an important role in tissue development and function. While its role is often complex, the shape and structure of microvascular networks are studied in conjunction with disease. Due to imaging constraints, these studies largely focus on the local network structure constrained to a limited field of view or tissue section. Recent advances in three-dimensional microscopy, including light sheet fluorescence microscopy (LSFM) (Kirst et al., 2020), knife-edge scanning microscopy (KESM) (Mayerich et al., 2008), and X-ray microtomography (µ-CT) (Hong et al., 2020; Quintana et al., 2019), overcome this limitation by enabling whole-organ imaging in small animals. However, the microscopy community currently lacks scalable algorithms and benchmarks to quantify microvascular structure at such a large scale.

While many algorithms exist for segmenting vessel-like structures, their scalability on modern whole-organ three-dimensional images has not been rigorously assessed. In addition, segmentation errors can increase disproportionately with volume coverage because acquiring larger volumes introduces trade-offs in SNR, resolution, and sampling anisotropy. This requires more complex algorithms - including pre-processing and machine learning - that are not as scalable as traditional thresholding or segmentation based on localized features (Daetwyler et al., 2019).

In this paper, we propose a comprehensive yet compact survey of available algorithms. We focus on essential features for microvascular analysis, including extracting vessel surfaces and the network’s associated connectivity. Algorithms were selected based on popularity and availability and provide a thorough quantitative analysis of their performance on datasets acquired using emerging techniques.

## 2 Microvascular models

Microvasculature is a meshwork of capillaries that penetrate tissue to provide nutrients and remove cellular waste. The structure of this mesh changes over time and plays a critical role in tissue function and disease progression. Most current studies are limited to small volumes, primarily characterizing vascular density, along with morphological metrics such as capillary length, radius, and tortuosity (Cassot et al., 2006). These metrics provide fundamental information about the network’s geometric properties and spatial organization. They also serve as the foundation for more advanced analyses, such as quantifying flow dynamics, assessing tissue perfusion, and understanding function. Moreover, downstream pipelines such as computational flow modeling and perfusion simulations can incorporate the segmentations and skeletons to enable quantitative studies of hemodynamics and tissue function (Vidotto et al., 2019).

**FIGURE 1:**
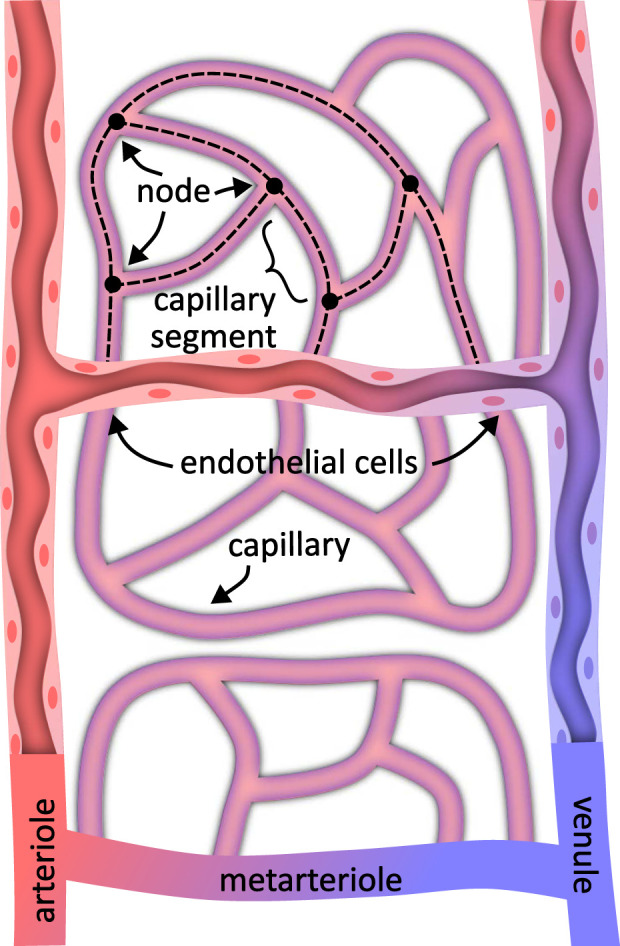
A microvascular model specifies both the *geometry* representing the surface structure and *connectivity* in the form of a spatial graph. Depending on the labeling method, the vascular geometry may represent the vessel interior or include endothelial cells lining the vessel and capillary walls. The vessel connectivity generally consists of a graph of vessel centerlines joined at nodes where vessels interconnect.

As image sizes increase, researchers attempt to apply existing metrics at larger scales using software packages like VesselVio (Kirst et al., 2020) or multi-step pipelines such as TubeMap. We expect new metrics and biomarkers to develop over time as larger models are explored. For now, we focus on methods that convert large-scale microvascular images into explicit models that support existing analyses. This leads to two key representations: the microvascular geometry and its skeleton (Figure 1).

**FIGURE 2:**
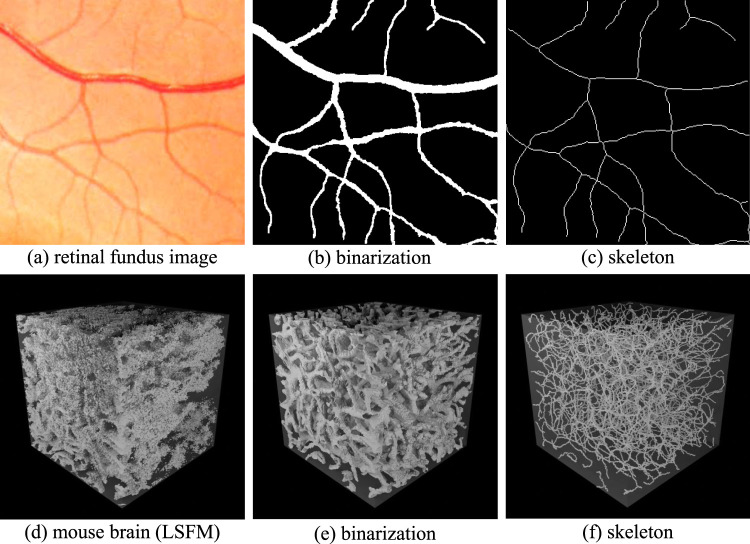
Implicit representations of binarization and skeletonization in 2D and 3D images. Retinal fundus images are shown in 2D **(a)**. The associated binarization **(b)** indicates pixels that lie inside (white) and outside (black) of the vascular network. The skeletonization **(c)** shows the vessel paths and points where they connect. The 3D case shows a volumetric visualization of an LSFM image **(d)** with the associated binarization **(e)** and centerlines **(f)**.

The geometry of the network represents the vascular surface that separates the region inside from the surrounding tissue. This representation enables the calculation of metrics such as vessel radii, surface area, and volume. The geometry is frequently extracted by identifying these inside/outside regions and then calculating the surface that separates them. Binarization is the first step for resolving the microvascular geometry (Figures 2b,e). These methods rely on separating pixels within the network from the surrounding tissue, providing an implicit representation of the geometry that can be readily converted to an explicit surface mesh.

The network skeleton represents its topology, combining a connectivity graph with vessel centerlines. This representation enables the calculation of metrics such as tortuosity, vessel length, and branching statistics. The skeleton is usually extracted from the geometry using specialized thinning algorithms. Skeletonization is used to extract vessel centerlines (Figures 2c,f) and calculate a connectivity graph. Skeletonization methods frequently rely on an initial binarized volume or surface mesh. However, some techniques, such as tracing, can compute the skeleton directly (Govyadinov et al., 2019).

### 2.1 Geometry

Vessel geometry can be represented using implicit (voxel-based) or explicit (mesh-based) data structures. In the implicit representation, the vascular network is segmented from surrounding tissue using a three-dimensional voxel grid similar to the original image, with individual voxels labeled as inside or outside vessels. Voxels are convenient for calculating volumes or performing voxel-wise statistical analyses.

Explicit representations, such as polygonal meshes, define vessel surfaces through interconnected vertices and edges. Meshes facilitate calculations of surface area and enable simulations requiring surface geometry. In this survey, all segmentation algorithms produce voxel-based results. We tested two skeletonization algorithms (**tagliasacchi** and **antiga**) that require triangular meshes as input, which we produce from segmentation results using the marching cubes algorithm.

This implicit representation can optionally be converted to a surface mesh using algorithms such as marching cubes (Lorensen and Cline, 1998). Measurements are taken across either structure as convenient. For example, surface area can be measured by integrating across a surface mesh, while volume can be measured by adding up the pixels within the network.

### 2.2 Skeleton

The vascular skeleton is almost always represented explicitly for analysis using connected curves. This explicit representation is fundamentally a connectivity graph, where each node represents a bifurcation and each edge represents a single non-branching vessel segment (Figure 1). The vessel centerlines are curves that can be integrated to calculate features such as length and tortuosity. Algorithms such as depth- and breadth-first searches can also be applied to calculate path lengths and branching characteristics.

**FIGURE 3:**
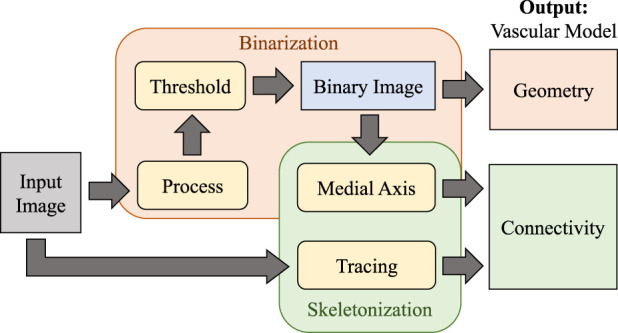
Overview of the vascular modeling pipeline. The input image is first processed (using various filtering or machine learning approaches) and binarized to extract geometry. The medial axis is then calculated to determine network connectivity. Tracing may also be applied directly to the raw image as an alternative approach for skeletonization.

In this paper, we evaluate the performance of algorithms for extracting the geometry and connectivity of a microvascular network in large images on imaging methods applicable to whole organs. Most algorithms first binarize the original image using semantic segmentation, using the result as the foundation for medial axis transforms that provide the skeleton (Figure 3).

## 3 Microvascular imaging

Microvascular imaging faces two competing challenges. First, imaging systems must be capable of collecting ≈ 1 mm^3^ of tissue to characterize network structure, with preferred volume coverages of 1 cm^3^ for entire rodent organs. Second, the imaging system must resolve microvessels that are less than 10 µm in diameter. Traditional techniques, such as widefield or confocal microscopy, are limited to two-dimensional sections or small volumes. The data in this paper were acquired using recent high-throughput techniques, including (1) X-ray microtomography (µ-CT), (2) light sheet fluorescence microscopy (LSFM), and milling microscopy (Mayerich et al., 2011; Guo et al., 2019).

### 3.1 Imaging methods

Meeting the criteria for resolution and volume coverage is challenging because microscopes are diffraction-limited and tissue samples are highly scattering. However, recent advances are starting to enable sufficient resolution and volume coverage. This paper considers the following broadly-accessible imaging methods:

X-ray microtomography (µ-CT) is nondestructive and measures the absorbance of X-rays incident on a sample to create three-dimensional images (Ritman, 2004). While µ-CT enables large-volume imaging of whole mouse brains (Hong et al., 2020), its low contrast limits spatial resolution. Recent advances in vascular perfusion compounds such as Vascupaint 2 (MediLumine, Montreal, Quebec, Canada) improve µ-CT resolution to ≈ 20 ± 4.0 µm, whereas previous contrast agents limited features to ≈92 ±  25 µm (Margolis et al., 2024).

**FIGURE 4:**
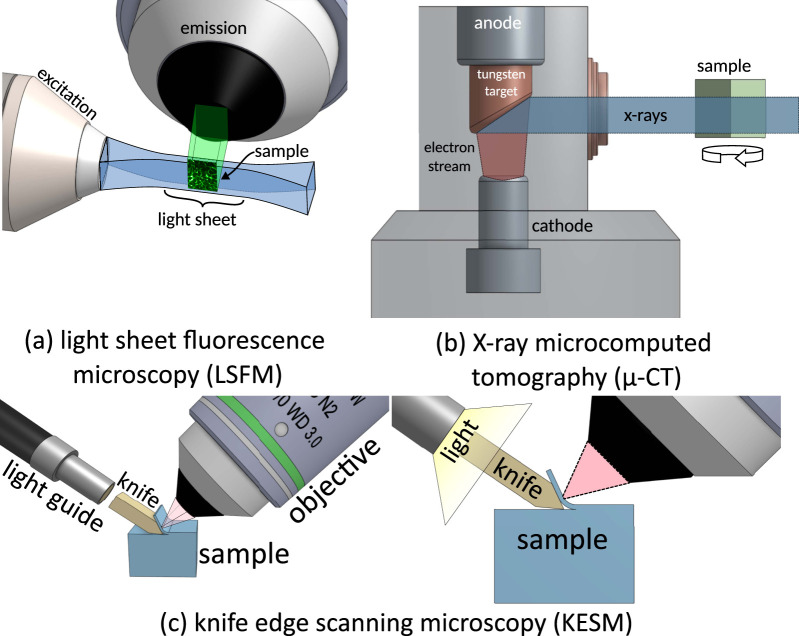
Imaging techniques tested for microvascular reconstruction. **(a)** Light sheet fluorescence microscopy uses a laser-scanned light sheet to excite fluorophores within a sample plane that are imaged through a high-NA objective. **(b)** X-ray micro-CT images the sample by rotating it within a transmission X-ray beam. **(c)** Knife-edge scanning microscopy is a milling-based imaging system that images tissue slices as they are sequentially ablated from a sample.

Light sheet fluorescence microscopy (LSFM) is characterized by separating illumination and detection. A thin sheet of light illuminates the sample (Hsu et al., 2022), and an orthogonally oriented objective (Figure 4) collects the emitted two-dimensional image using a CCD or CMOS camera. While the penetration depth is traditionally limited by tissue scattering, recent developments in clearing protocols (ex. CUBIC or iDISCO+) (Susaki et al., 2015; Renier et al., 2014) enable large-scale imaging of whole rodent brains. Recent cleared-tissue implementations span a broad range of resolutions. For example, hybrid open-top light sheet systems can achieve lateral resolution of 0.45 µm and axial resolution of 2.9 µm across millimeter-scale volumes (Glaser et al., 2022). When combined with 4× expansion microscopy, LSFM has reached effective resolutions of 375 nm laterally and 750 nm axially in centimeter-scale samples (Glaser et al., 2025). More recently, axially swept LSFM designs have demonstrated nearly isotropic resolution of approximately 300 nm in fixed and cleared tissues (Lin et al., 2025). These gains in resolution typically trade off against imaging volume coverage or acquisition speed, so whole-organ datasets still exhibit significant anisotropy.

Milling microscopy removes layers of a sample during the imaging process to expose deeper tissue volumes. Initial experiments used on two-photon microscopy followed by photo-ablation (Tsai et al., 2003), demonstrating that volume constraints could be eliminated by systematically removing tissue. More recent techniques separate tissue sections using physical cutting. Knife-edge scanning microscopy (KESM) (Mayerich et al., 2008; Mayerich et al., 2011) separates a tissue slice from the rest of the block during imaging, while milling with ultraviolet surface excitation (MUSE) performs block-face imaging followed by ablation (Guo et al., 2019).

### 3.2 Datasets

We evaluated our binarization and skeletonization methods on three datasets representing the modalities described in Section 3.1.

**FIGURE 5:**
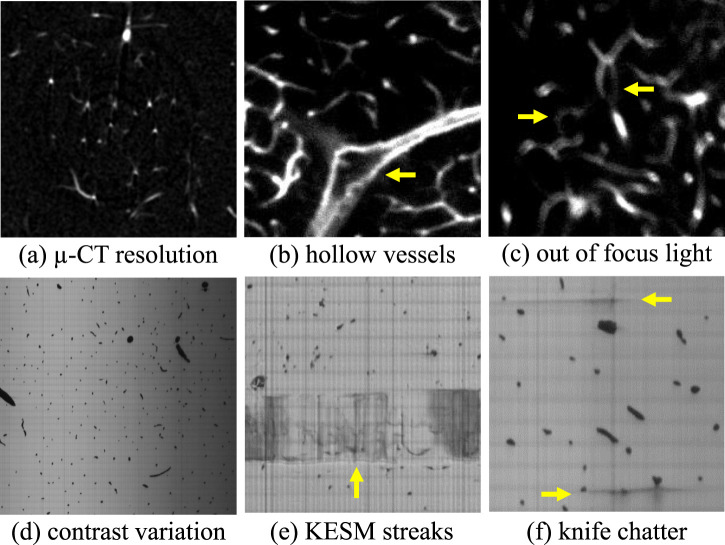
Noise and systematic artifacts that occur in high-throughput imaging techniques. Limitations in resolution **(a)** reduced the ability to detect small vessels that form connections in the network. Large vessels in LSFM are often hollow **(b)** because contrast is provided by labeling the vessel wall. Misalignments during imaging can also produce blurry sections **(c)** that can confound segmentation algorithms. KESM introduces physical artifacts such as variations in illumination across a slice **(d)** and physical streaks caused by the interaction between the sample and cutting tool **(e,f)**.

X-ray microtomography (**µ-CT**) scans of mouse brain (*Tg(Slco1c1-BAC-CreER)*; *R26* ^*-lsl-TdTom/+*^) vascular networks were acquired using a Skyscan 1276 (Bruker, Billerica, MA, United States) at an isotropic sampling rate of 10 µm per voxel. Mice were prepared based on previously published protocols (Suarez et al., 2024). Briefly, the vasculature was perfused with Vascupaint (MediLumine, Product number: MDL-121; Montreal, Quebec, Canada) to provide vascular and microvascular X-ray contrast (Figure 5a).

Light sheet fluorescence microscopy (**LSFM**) images of mouse brain microvasculature were acquired using a Cleared Tissue LightSheet (CTLS) Microscopy Workstation XL (3i, Intelligent Imaging Innovations, Denver, CO), equipped with a 30 fps Fusion BT sCMOS camera ( 2304×2304 resolution, 6.5 µm pixel size, 95% quantum efficiency, cooled to −50 °C; Hamamatsu Photonics, Japan). Adult C57BL/6N female mice (4.5 months old) were perfused with fluorescently labeled lectin (488 nm; Vector Laboratories, DL-1174-1) as previously described (Ahn et al., 2024), and brains were optically cleared using the iDISCO protocol (Hsu et al., 2022). Imaging was performed using a 1X (0.25 NA) objective and a 488 nm laser at 200 mW power with a 300 ms exposure time. Image stacks were acquired at a 6 µm step size in the Z-direction, with 15% right-side overlap and 50% center tile overlap, making the voxel size 2.0×2.0 × 6.0 µm. Images were stitched and reconstructed using SlideBook^™^ software (Intelligent Imaging Innovations) with the LightSheet module for 3D multipoint acquisition. Raw image files were acquired in 16-bit TIFF format and rescaled to 8-bit using ImageJ for downstream processing. Final voxel spacing was adjusted to 1.0 × 1.0 × 0.75 µm using linear interpolation.

Milling microscopy images were acquired using a knife edge scanning microscope **KESM** at a voxel resolution of 0.6×0.7×1 µm, covering a 0.6×0.6×2 mm volume. The tissue was acquired from a normal mouse (C57BL/6J) perfused with India ink (Mayerich et al., 2011). The entire dataset is available using the KESM Mouse Brain Atlas (Chung et al., 2011).

Ground truth volumes for training and validation were manually annotated in Slicer (Kikinis et al., 2013). Binarization and skeletonization methods were evaluated on a 200×200×200 voxel dataset. The machine learning models were trained on six separate 128×128×128 voxel sub-volumes for each dataset (distinct from the $200^{3}$ sample used for evaluation).

### 3.3 Noise and artifacts

Each of these imaging modalities introduces sources of noise and other artifacts that challenge segmentation, including:

Resolution limitations are introduced due to both the signal strength and diffraction limit. Standard LSFM is typically anisotropic, with poorer axial than lateral resolution. Recent implementations (Glaser et al., 2022; Lin et al., 2025) can achieve sub-micron isotropic resolution, with trade-offs in speed or volume coverage. µ-CT is primarily limited by SNR to ≈ 20 µm in all three dimensions. Both of these constraints are larger than the diameter of the smallest microvessels (Figure 5a).

Staining and labeling used in LSFM relies on targeting cells in the vessel wall, producing hollow vessels when their diameter is significantly larger than the diffraction limit (Figure 5b). In practice, vascular perfusion with fluorescent compounds like dextran can overcome these artifacts while improving contrast (at the expense of molecular specificity). In that case we would expect images that are more comparable to KESM.

Blurred vessels can occur in LSFM images due to temporary misalignment during long imaging periods (Figure 5c).

Contrast variations are introduced by non-uniform illumination and/or staining in both LSFM and KESM. Contrast is also reduced in LSFM as a function of depth due to scattering.

Machining artifacts introduced by cutting tools in milling microscopy can cause lines (Figure 5e) and streaks (Figure 5f) in individual z-axis slices.

## 4 Evaluation methodology

We characterize each algorithm’s performance using established metrics and perform an evaluation of adjustable parameters. All algorithms were tested on 200×200× 200 voxel volumes that were manually segmented to create an implicit representation of the geometry and skeleton. If an algorithm requires optimization (ex. U-Net), training is performed on a separate volume acquired from an independent dataset. The same training and validation sets are used for all algorithms.

### 4.1 Selection criteria

We evaluate a subset of available algorithms based on multiple criteria, prioritizing algorithms used in popular, domain-specific vessel analysis software. This includes Slicer (Kikinis et al., 2013), the Vascular Modeling Toolkit (VMTK) (Izzo et al., 2018), and VesselVio (Bumgarner and Nelson, 2022). Second, we prioritized algorithms with an established open-source implementation that was preferably provided by the authors. We selected representative algorithms from three classes of methods, including: (1) classical thresholding methods, (2) Hessian-based and gradient-based enhancement filters, and (3) deep learning-based semantic segmentation models. We selected skeletonization methods for a range of input data types, including: (1) binarized images, (2) meshes, and (3) point clouds. Our goal is to reflect diverse methodological approaches used for microvascular modeling. This selection process ensures that our evaluation adequately represents current best practices and popular trends in the field, providing a robust benchmark for future developments. Table 1 provides the dates and citations of the proposed algorithms.

**TABLE 1:** Algorithms assessed in this paper for performing binarization (left) and skeletonization (right). The publication date is shown along with their citation count as of this writing.

Segmentation	
Method (year)	Citations
otsu (1979)	58,569
frangi (1998)	5,580
oof (2008)	316
bfrangi (2015)	346
unet (2015)	116,836
nnunet (2021)	6,480

Skeletonization	
lee (1994)	2032
palagyi (1999)	275
antiga (2003)	62
kline (2010)	52
tagliasacchi (2012)	316
kerautret (2016)	11

Otsu’s method was evaluated as a baseline binarization algorithm (**otsu3d**), and used as the final binarization step as required for other algorithms. Vesselness filters, including both Frangi (**frangi**) and Beyond Frangi (**bfrangi**), were included due to their overwhelming popularity for vessel enhancement. Optimally oriented flux (**oof**) filters were included as a more recent innovation with the open-source implementations provided by the authors. U-Net architectures are extensively used for semantic segmentation in biomedical imaging, so we elected to test a baseline U-Net architecture (unet) while including recent work on self-configured U-Nets (**nnunet**).

We selected the thinning algorithms by Lee (**lee**) and Palágyi (**palagyi**) because of their popularity in skeletonization literature. While the level-set method by Kline (**kline**) and the confidence accumulation method by Kerautret (kerautret) are not used in existing modeling packages, the authors provide open-source implementations that were tested. We also selected the most recent skeletonization methods available for meshes (**tagliasacchi**). The selected methods and keywords are provided in Table 2.

**TABLE 2:** Algorithms evaluated in this paper, along with their classes and output data types. Algorithm types are shown in the first column alongside names used to reference the associated results. Remaining columns show the algorithm output (geometry or skeleton). Preprocessing methods such as “vesselness” filters and OOF are used to enhance vessels prior to thresholding with Otsu’s method.

Algorithm	Produces		
	Geometry	Skeleton	Preprocessing
Otsu’s Method (otsu3d)	**X**		
Vesselness filter (frangi, bfrangi)			**X**
Optimally Oriented Flux (oof)			**X**
Machine Learning (unet, nnunet)	**X**		**X**
Thinning (lee, palagyi)		**X**	
Gradient-Based (kerautret, kline)		**X**	
Mesh-Based (tagliasacchi, antiga)		**X**	

### 4.2 Geometry metric

Since microvasculature accounts for ≈ 4% of the total tissue volume, we focus on metrics that can accurately characterize unbalanced classifications. The Jaccard similarity index (J) and Dice similarity coefficient (D) are the most common for binarization. The Jaccard index is the normalized volume overlap between the binarization and ground truth:

$$J\left(A,B\right)=\frac{\sum \left(A\cap B\right)}{\sum \left(A\cup B\right)}$$

The Jaccard index is in the range J∈[0,1] , where J=0 indicates no overlap and J=1 indicates that the binarization is identical to the ground truth.

We also calculate the precision, emphasizing the accuracy of the positive predictions, and recall, which quantifies the model’s ability to identify true positives. The precision (p) and recall (r) are often combined using the F-score (F), which is both equal to the Dice coefficient and a function of the Jaccard index in the case of binarization:

$$F=\frac{2pr}{p+r}=D=\frac{2J}{1+J}$$

### 4.3 Skeleton metric

We adopt the definition of the skeleton as: a set of curves that have identical topology to the geometric surface and are equidistant to the boundary (Wei et al., 2018). We enforce these conditions by manually generating ground truth skeletons based on this definition.

Skeletonization is evaluated using NetMets (Mayerich et al., 2012), which calculates the similarity between two sets of curves A and B in 3D space:

$$M\left(A,B\right)=\frac{1}{L}\underset{a\in A}{\sum }\int e^{\frac{d{\left[a\left(t\right),B\right]}^{2}}{2\sigma^{2}}}dt$$

where L is the total length of all curves in the set A , a(t) is a point on the i th curve parameterized by t , and σ is a sensitivity parameter. The function d(x,B) is the distance between a point x and the closest point in the set of curves B . Given two networks representing the set of curves in the ground truth G and test case T , the precision (positive predictive value) P and recall (true positive rate) R are calculated:

$$P=M\left(T,G\right)\;R=M\left(G,T\right)$$

The precision is the percentage of the test skeleton that correctly corresponds to the ground truth, and the recall is the percentage of the ground truth skeleton that is correctly detected in the test case. The sensitivity parameter σ=1 µm is used for all images.

## 5 Segmentation algorithms and evaluation

The selected segmentation algorithms largely fall into three groups that largely build on each other: (1) basic thresholding, (2) vessel enhancement and preprocessing, (3) convolutional neural networks.

Thresholding is the most basic approach to binarization, and in many cases an optimal threshold can be calculated automatically. Minimum cross entropy (Li and Tam, 1998), IsoData (Ridler and Calvard, 1978), and Fuzzy thresholding (Huang and Wang, 1995) are popular approaches (Kramer et al., 2022; Huang et al., 2019) that exist in several software packages (Fedorov et al., 2012). The most established algorithm is Otsu’s method, which determines the optimal threshold to separate foreground and background components.

Modern approaches rely on some form of image enhancement that is applied prior to thresholding. This includes filters designed to enhance the contrast of tube-like structures, as well as machine learning to perform semantic segmentation. The methods tested here include vesselness filters (Frangi et al., 1998; Jerman et al., 2016) and optimally oriented flux (OOF) (Law and Chung, 2008).

Convolutional neural networks (CNNs) have taken a prominent role in image segmentation. The U-Net architecture (Ronneberger et al., 2015) and its self-configuring extension, nnU-Net (Isensee et al., 2021), represent the current state-of-the-art in semantic (pixel-level) segmentation. These architectures use a symmetric U-shaped encoder-decoder design (Figure 10) to capture details at multiple scales by processing progressively larger image patches. While machine learning approaches tend to outperform deterministic methods, a significant amount of effort must be applied to annotation and training. As a result, the deterministic approaches are still extensively used in popular software packages.

All methods that require thresholding use Otsu’s method (Otsu, 1979), which computes a threshold θ that maximizes the between-class variance. Otsu’s method was also tested alone (**otsu3d**) as a baseline binarization method.

Several preprocessing methods use scale-space filtering (Witkin, 1987) to account for variations in vessel diameter. Scale-space approaches add a discrete dimension to a field (ex. I(x)→I(x,s) ) that represents feature sizes. The new dimension is based on a pre-selected set of scale-space parameters $\Sigma \in \left[\sigma_{0},\sigma_{1},\ldots ,\sigma_{S-1}\right]$ . The same scale range was used for **frangi**, **ofrangi**, and **bfrangi**. However, the **OOF** method used slightly smaller values to reach better performance.

### 5.1 Hessian-based vessel enhancement

The most popular method for enhancing vessels is the Hessian-based approach described by Frangi et al. (1998), originally designed for 2D retinal fundus images. The Hessian matrix is calculated at each point in the image $x\in R^{3}$ using finite differences across S scales, creating a field $H\left(x,s\right)\in R^{3\times 3}$ . The filter response V(x)∈R is calculated using the Hessian matrix eigenvalues $\left(\left|\lambda_{1}|\leq |\lambda_{2}|\leq |\lambda_{3}\right|\right)$ (Supplementary Algorithm S1). In this study, the method introduced by Yang and Cheng (2014) was also used to accelerate the computations of the Hessian matrix by a factor of two.

**FIGURE 6:**
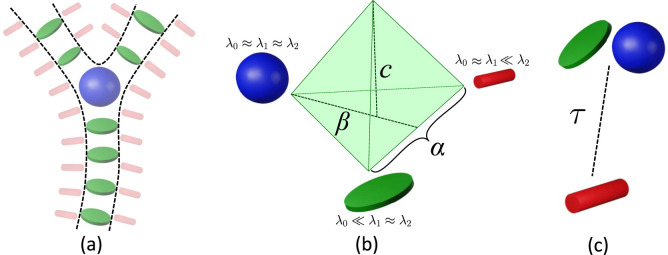
Hessian-based filters emphasize **(a)** plate-like (green) and blob-like (blue) tensors as vessels and bifurcations, while stick-like (red) tensors emphasize surfaces (including vessel surfaces). **(b)** The vesselness filter proposed (frangi, ofrangi) uses parameters α , β , and c to target voxels associated with plate-like and blob-like features. α is used to discern between plate-like and line-like structures, β emphasizes the deviation from a blob-like structure, and c controls the Frobenius norm of the Hessian matrix and suppresses the noise. **(c)** bfrangi simplifies these tunable parameters into a single value τ to differentiate between vessels and background (composed of small and stick-like tensors).

**FIGURE 7:**
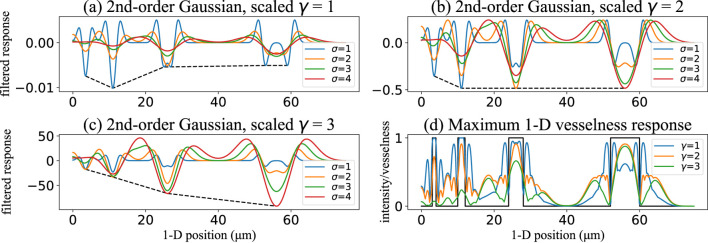
Effect of the scale-normalization exponent γ on synthetic one-dimensional vessel cross-sections. The second-order Gaussian derivatives are shown alongside the resulting vesselness response. **(a–c)** Second derivative responses to a 1D signal convolved with scale-normalized Gaussian second derivatives at multiple scales ( σ = 1, 2, 3, 4), plotted for different normalization exponents: **(a)** γ = 1, **(b)** γ = 2, and **(c)** γ = 3. **(d)** Maximum 1D vesselness response (across all scales) for γ = 1, 2, and 3. Only γ = 2 yields consistent, scale-invariant responses.

The original “vesselness” algorithm (**frangi**) is outlined in Algorithm 1 and relies on four parameters: tuning parameters α , β , c that target the vessel shape, and a scale parameter γ≈2 . The shape parameters are balanced to separate tube-like structures and bifurcations from background pixels (Figure 6). The scalar γ was proposed earlier (Lindeberg, 1998) to tune the derivatives used in the Hessian. Attempts to optimize this parameter yield a consistent value of γ=2 in the literature. We found that this is due to the scale factor $\sigma^{\gamma }$ compensating for energy dissipation from the second-order Gaussian scale-space filter. A value of γ=2 ensures that the most intense response comes from features near the scale-space parameter σ (Figure 7).

**FIGURE 8:**
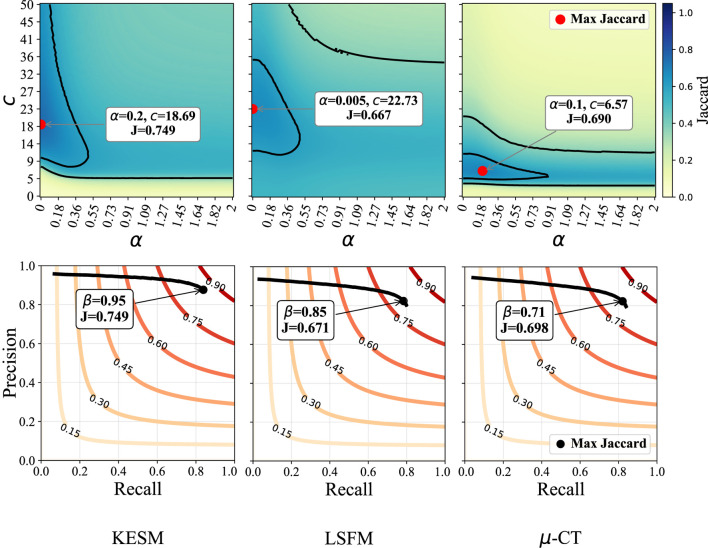
Sensitivity to α , β , and c parameters in **frangi** method across the three datasets. Top row: Jaccard index (color-mapped) as a function of α and c parameters, with β tuning factor held constant (effectively removed, i.e., set to 1). The red dot indicates the parameter combination yielding the maximum Jaccard score for each dataset. Bottom row: Jaccard index (contours) as a function of precision and recall for varying β values, with α and c fixed at their optimal values identified in the top row. The black dot marks the point of maximum Jaccard score, and the continuous line shows the score as β varies.

Since the parameters in the original algorithm can be challenging to select, we provide two comparisons. First, we examine binarization results based on parameters used in the original paper (**frangi**), and after parameter optimization (**ofrangi**) for each dataset using training data. A parallel implementation was used to create sensitivity maps for each parameter (Figure 8). The relationship between parameters for **frangi** is shown in Figure 6a.

**FIGURE 9:**
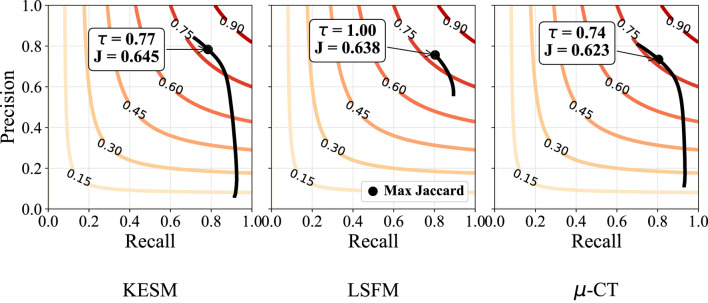
Sensitivity to τ in the Beyond Frangi method for each dataset. Red curves represent contours where the Jaccard index is constant. Precision-recall curves show the change of both metrics in response to varying τ . The indicated point is the maximum Jaccard index, corresponding to the best parameter for τ using this metric.

A modified implementation of the vesselness framework replaces the parameters with a single normalization term τ (Jerman et al., 2015). The Beyond Frangi (**bfrangi**) algorithm (Supplementary Algorithm S2) regularizes the largest eigenvalue at each scale, yielding a more uniform response across vessel cross-sections and intensities. The sensitivity of each dataset to this parameter is illustrated in Figure 9.

### 5.2 Optimally oriented flux

The second derivatives within the Hessian are numerically calculated using finite-difference methods, introducing artifacts when features are adjacent. An alternative approach proposes optimally oriented flux (OOF) (Law and Chung, 2010), which relies on first derivatives and integrating gradient information over a spherical region. This applies a hard cut-off that localizes the gradient information used.

**FIGURE 10:**
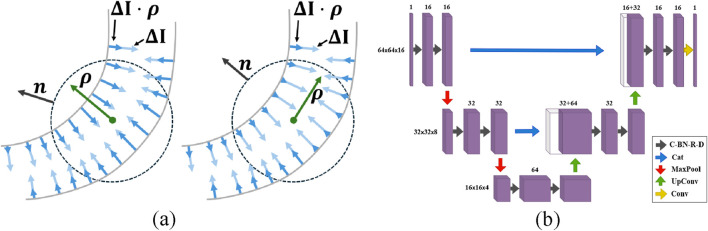
In Optimally Oriented Flux **(a)**, the vessel orientation is determined by maximizing the outward-oriented gradient flux over a spherical region. Diagram of the U-Net architecture **(b)**. The network consists of convolutional layers with batch normalization, ReLU activation, and dropout (C-BN-R-D), concatenation layers (Cat), max-pooling layers (MaxPool), up-convolutional layers (UpConv), and convolutional layers (Conv).

Given a point x in the 3D image I(x)∈R and a direction vector ρ , the gradient flux aligned with ρ is integrated over a sphere Ω with radius $\sigma_{s}$ (Figure 10).

$$F\left(x,r,\rho \right)=\iint_{\Omega }\left(\nabla I\left(x\right)\cdot \rho \right)\rho \cdot ndA=\rho^{T}Q\left(x,r\right)\rho$$

where dA is the differential area of the sphere and n is the sphere normal at x . This integral is used to calculate the flux matrix Q . The optimal vessel orientation is the vector ρ that maximizes the outward-oriented flux:

$$\underset{\rho }{\max}\left[\rho^{T}Q\left(x,r\right)\rho \right]$$

Our tests used the OOF method available in MATLAB (**oof**), where the only tunable parameters are the sphere radii Σ . In our tests, we use the same Σ values for OOF and scale-space arguments (**frangi**, **ofrangi**, **bfrangi**).

### 5.3 Convolutional neural networks

The U-Net (unet) architecture uses parallel encoder/decoder paths bridged by skip connections that merge high-resolution features from the encoder into the decoder. The network meta-parameters were determined through iterative trial and error based on validation performance. This U-Net uses 3 × 3 × 3 convolutional layers, ReLU activation, and 2 × 2 × 2 max-pooling layers (Figure 10). The data is divided into smaller volumes, selecting only those with intensities above the global mean to avoid empty or near-empty voxels that reduce training quality (Çiçek et al., 2016). The unet model tested here is trained and tested on these selected volumes using the Focal Tversky loss function (Abraham and Khan, 2019).

The nnU-Net (nnunet) architecture builds on U-Net by overcoming the need for manual meta-parameter tuning. nnU-Net maintains the encoder-decoder structure with skip connections while automatically adapting its architecture and hyperparameters. Like U-Net, it employs 3 × 3 × 3 convolutional layers, ReLU activation, and pooling layers but integrates additional strategies, such as ensemble modeling and deep learning-based boundary enhancement to improve segmentation accuracy (Schott et al., 2023). During training, nnU-Net uses standard loss functions and ensemble modeling, where multiple models are trained and combined. We used the source code provided by the authors^1^ for these experiments.

### 5.4 Geometry results

**FIGURE 11:**
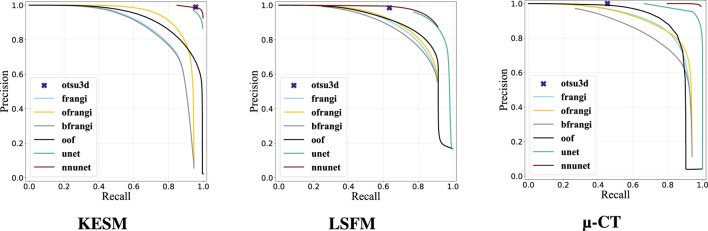
Precision-Recall curve of evaluated segmentation methods.

Our binarization results are shown in Table 3, providing the Jaccard Index, precision, and recall. Otsu’s method (otsu) was tested on raw data and used to select the final threshold for all preprocessing algorithms. The vesselness filter was tested using published parameters in the original paper (**frangi**) and after optimization of all parameters (**ofrangi**), while Beyond Frangi (**bfrangi**) was tested after optimization of τ . Optimally oriented flux (**oof**) does not require parameter tuning outside of the scale-space parameters used to select sphere radii. Precision-recall curves are provided for the raw output so that the reader can evaluate the results without Otsu’s method (Figure 11). Like most semantic segmentation networks, unet and nnunet integrate binarization into the model and do not require Otsu’s method for thresholding.

**TABLE 3:** Segmentation results showing the Jaccard index, precision, and recall for each binarization algorithm (red = bad, green = good). The original vesselness paper (frangi) recommends 0.5, 0.5, and half of the maximum value of the Hessian norm for α , β , and c , respectively. The standard deviation (STD) values are computed as part of a statistical test across three non-overlapping volumes of equal size.

Binarization methods								
		otsu3d	frangi	ofrangi	bfrangi	oof	unet	nnunet
KESM	Jaccard Index	0.946 ± 0.030	0.403 ± 0.015	0.749 ± 0.005	0.645 ± 0.013	0.713 ± 0.017	0.904 ± 0.016	0.952 ± 0.010
	Precision	0.989 ± 0.016	0.985 ± 0.001	0.877 ± 0.017	0.777 ± 0.030	0.828 ± 0.025	0.912 ± 0.014	0.990 ± 0.011
	Recall	0.956 ± 0.022	0.405 ± 0.015	0.837 ± 0.020	0.791 ± 0.011	0.837 ± 0.043	0.989 ± 0.003	0.961 ± 0.001
LSFM	Jaccard Index	0.628 ± 0.151	0.183 ± 0.054	0.671 ± 0.063	0.638 ± 0.037	0.696 ± 0.069	0.804 ± 0.013	0.764 ± 0.117
	Precision	0.984 ± 0.035	0.998 ± 0.000	0.822 ± 0.021	0.756 ± 0.058	0.806 ± 0.133	0.875 ± 0.013	0.969 ± 0.014
	Recall	0.635 ± 0.188	0.184 ± 0.054	0.786 ± 0.070	0.803 ± 0.019	0.837 ± 0.040	0.908 ± 0.003	0.783 ± 0.109
μ **-CT**	Jaccard Index	0.454 ± 0.021	0.054 ± 0.003	0.698 ± 0.020	0.623 ± 0.034	0.724 ± 0.026	0.819 ± 0.013	0.963 ± 0.037
	Precision	1.00 ± 0.000	1.00 ± 0.000	0.821 ± 0.004	0.734 ± 0.013	0.880 ± 0.013	0.822 ± 0.012	0.991 ± 0.014
	Recall	0.454 ± 0.021	0.054 ± 0.003	0.823 ± 0.025	0.804 ± 0.039	0.803 ± 0.029	0.996 ± 0.003	0.966 ± 0.047

**FIGURE 12:**
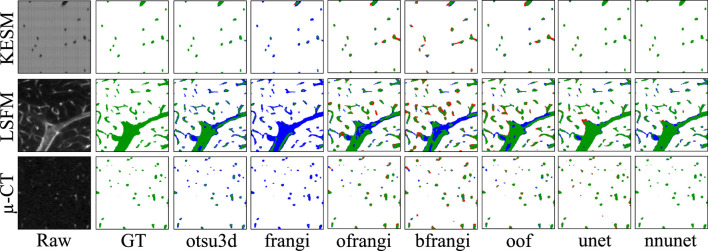
Cross-sections of three-dimensional volumes showing segmentation results for each modality. Green pixels indicate correctly labeled vessels, while white pixels show correctly detected background. Blue indicates undetected vessels (false negatives), while red indicates background pixels that are incorrectly labeled as vessels (false positives). GT indicates the manually annotated ground truth.

Raw image sections are provided from each dataset, along with the best corresponding binarization result for each algorithm (Figure 12). Further discussion is provided in Section 7.

## 6 Skeletonization algorithms and evaluation

The presented skeletonization algorithms require either an initial binary image or a geometric surface. Whenever the vascular surface is required, it is extracted using the marching cubes algorithm from the binarized output.

### 6.1 Skeletonization

Skeletonization is the method of determining the connected vessel centerlines, usually based on the extracted geometry. A binarized image is used as an input to thinning algorithms, while other approaches calculate the skeleton (or medial axis) from an explicit geometry.

Thinning uses morphological erosion to calculate an implicit representation of the skeleton from a binarized image. Voxels are iteratively removed until a thin single-voxel skeleton remains. The concept was originally proposed in Blum’s grassfire propagation (Blum, 1967), while Tsao and Fu (1981) were the first to introduce a three-dimensional algorithm based on formalized topological and geometric rules. The main concept in erosion-based methods is determining the “simple points” that can be removed without changing the model topology. Thinning algorithms usually differ in how they detect and remove simple points. The most popular method used in current software packages (Fedorov et al., 2012; Bumgarner and Nelson, 2022) is Lee et al. (1994) approach.

Mesh-based medial axis transforms approximate the skeleton from a geometric surface. These include point-sampling methods that leverage ray casting Kerautret et al. (2016) to localize the skeleton. While these methods face challenges when differentiating the “inside” and “outside” of complex shapes (Wei et al., 2018), statistical methods can overcome these problems using vector accumulation maps to probabilistically approximate the skeleton (Kerautret et al., 2016). This can be overcome by directly evolving the geometry towards its centerline using an active contour model (Tagliasacchi et al., 2012).

Another subcategory of mesh-based transforms leverages a Voronoi diagram of the surface (Antiga et al., 2003). The boundaries of the Voronoi cells within vessels can be tracked to extract the medial axis. Despite their precise results, Voronoi-based techniques require careful handling of boundary conditions and specifying seed points to avoid generating spurious branches.

### 6.2 Thinning

Thinning algorithms reduce an object to a single-voxel-thick centerline while maintaining topology (ex. connectivity, cavities, tunnels). This is accomplished by examining the local neighborhood of each voxel to identify simple points whose deletion preserves topology. These points are iteratively removed until a thinned skeleton remains.

The **lee** algorithm (Lee et al., 1994) identifies simple points using an octree to recursively subdivide the local neighborhood (Supplementary Algorithm S3). A later approach (Palágyi and Kuba, 1999) uses predefined templates to detect and flag simple points, which is easier to parallelize. Both the sequential **lee** and parallel palagyi algorithms are parameter-free and take a binarized image as input.

### 6.3 Gradient-based skeletonization

The Kerautret et al. (2015) algorithm extracts a centerline from a surface mesh by casting rays from each surface point along its normal. The rays are accumulated in a voxel grid where the maximal ridges represent centerlines. The most recent modification (Kerautret et al., 2016) determines an accumulation confidence value for each voxel, where points with higher confidence are more likely to be located on the centerline.

The only required parameter is the accumulation distance $\left(d_{\mathrm{acc}}\right)$ or ray length, which is slightly larger than the maximal radius of the input shape. This value is straightforward to approximate for each volume using the radius of the larger vessels.

The **kline** algorithm (Kline et al., 2010) calculates a distance field using the fast marching method (FMM), and then internal ridges (maxima) are followed from user-specified seed points. This algorithm assumes that the geometry is a connected tree of tubular structures branching from a single root point. Since microvascular networks do not have a unique root, a large number of seed points are required to comprehensively trace the network.

### 6.4 Mesh-based skeletonization

**FIGURE 13:**
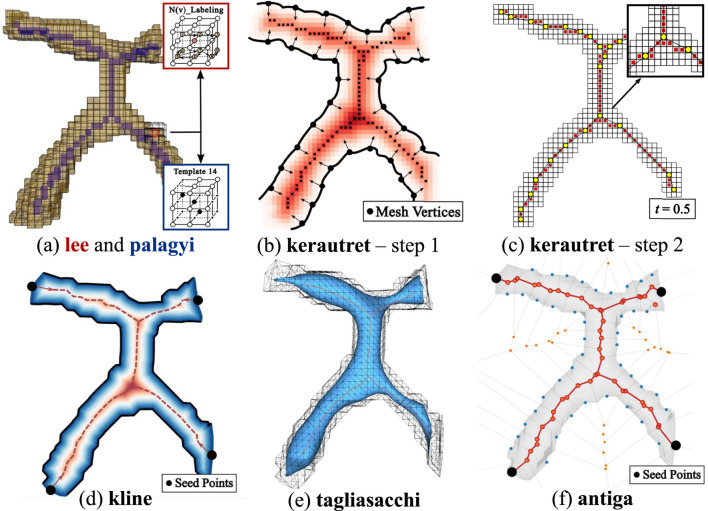
Overview of skeletonization methods. **(a)** Thinning-based methods: lee applies N(v)_labeling (red), and palagyi applies templates (blue) to detect simple points. **(b,c)** Kerautret generates a confidence map and extracts the centerline using morphological operations, geodesic segmentation, and FMM. **(d)** Kline computes a distance field and applies FMM to generate the skeleton through seed points. **(e)** Tagliasacchi contracts the input mesh iteratively. **(f)** Antiga constructs a Voronoi diagram to extract centerlines using seed points.

The tagliasacchi algorithm (Tagliasacchi et al., 2012) calculates the skeleton from a closed mesh using mean curvature flow (MCF). The medial axis is extracted by moving the mesh along its surface normal proportional to the surface curvature. This iteratively collapses the mesh into a one-dimensional curve (Figure 13). We tested the **tagliasacchi** method using an implementation provided by StarLab^2^, which requires a watertight manifold mesh and is highly sensitive to mesh boundaries. We forced a watertight mesh at all boundaries and applied the algorithm to each closed mesh component separately.

The antiga algorithm (Antiga et al., 2003) builds a Voronoi diagram from point samples of the vascular surface. The intersections of each Voronoi region lie equidistant to the sampled surface, such that medial axis curves can be traced between user-specified seed points. The Delaunay tessellation is calculated from a set of points P that densely samples the vascular surface, which provides the vertices V for the Voronoi diagram. The user specifies a set of seed points S that are connected by selecting points in V that maximize the distance from the vascular surface. This is done by solving an Eikonal equation using the fast sweeping method, ensuring a maximum distance from the boundary surface and providing stability in complex regions like bifurcations. This study uses the implementation of **antiga** provided in Slicer^3^. However, the method failed to process seed points or centerlines for messy surfaces created in some LSFM datasets (Table 4).

**TABLE 4:** Results of centerline extraction on binarization results (left columns: precision, right columns: recall). Thinning was performed using all binarized data for each dataset (see Table 3).

Skeletonization														
**Binarization**			**lee**		**palagyi**		**kerautret**		**kline**		**tagliasacchi**		**antiga**	
			P	R	P	R	P	R	P	R	P	R	P	R
	KESM	**otsu3d**	0.985	0.991	0.967	0.987	0.856	0.898	0.598	0.704	0.94	0.689	0.995	0.934
		**ofrangi**	0.992	0.975	0.98	0.982	0.875	0.891	0.584	0.707	0.944	0.969	0.996	0.955
		**bfrangi**	0.979	0.985	0.967	0.987	0.872	0.897	0.576	0.743	0.946	0.694	0.997	0.939
		**oof**	0.974	0.959	0.953	0.957	0.837	0.868	0.549	0.633	0.928	0.676	0.977	0.838
		**unet**	0.986	0.988	0.971	0.984	0.859	0.896	0.54	0.644	0.937	0.695	0.993	0.957
		**nnunet**	0.991	0.987	0.975	0.985	0.854	0.887	0.574	0.681	0.931	0.699	0.983	0.956
	LSFM	**otsu3d**	0.873	0.841	0.882	0.852	0.647	0.62	0.581	0.518	0.884	0.354	-	-
		**ofrangi**	0.948	0.799	0.925	0.792	0.533	0.533	0.505	0.417	0.66	0.182	0.908	0.388
		**bfrangi**	0.915	0.881	0.893	0.857	0.485	0.583	0.473	0.582	0.531	0.221	0.853	0.48
		**oof**	0.884	0.795	0.863	0.783	0.432	0.514	0.427	0.459	0.786	0.386	0.873	0.336
		**unet**	0.859	0.923	0.85	0.902	0.461	0.61	0.503	0.588	0.836	0.565	0.913	0.39
		**nnunet**	0.856	0.895	0.896	0.894	0.6	0.64	0.552	0.574	0.886	0.612	0.721	0.4
	µ-CT	**otsu3d**	0.963	0.821	0.959	0.857	0.791	0.466	0.69	0.481	0.912	0.55	0.994	0.73
		**ofrangi**	0.914	0.938	0.893	0.948	0.69	0.483	0.519	0.556	0.848	0.574	0.978	0.821
		**bfrangi**	0.919	0.916	0.896	0.931	0.659	0.489	0.45	0.506	0.854	0.574	0.981	0.852
		**oof**	0.852	0.913	0.831	0.937	0.622	0.385	0.537	0.577	0.821	0.603	0.986	0.821
		**unet**	0.832	0.967	0.792	0.969	0.503	0.284	0.473	0.576	0.808	0.581	0.984	0.937
		**nnunet**	0.889	0.966	0.855	0.971	0.6	0.384	0.562	0.619	0.808	0.591	0.971	0.908

### 6.5 Skeletonization results

The precision and recall for the proposed skeletonization methods are shown in Table 4. The NetMets algorithm is used to quantify performance using σ=1 µm. The results are provided using all binarization algorithms as input. The ground truth binarization is also provided as a baseline to demonstrate skeletonization from an “ideal” starting point (Table 5).

**TABLE 5:** Results for skeletonization on the ground truth segmentations.

Skeletonization													
		lee		palagyi		kerautret		kline		tagliasacchi		antiga	
		P	R	P	R	P	R	P	R	P	R	P	R
GT	KESM	0.994	0.991	0.974	0.987	0.858	0.895	0.591	0.714	0.938	0.698	0.995	0.951
	LSFM	0.919	0.994	0.912	0.962	0.510	0.653	0.534	0.744	0.817	0.625	0.934	0.475
	µ-CT	0.874	0.968	0.840	0.969	0.603	0.429	0.559	0.650	0.796	0.584	0.987	0.951

The **lee** and **palagyi** methods achieved the highest F-score across all images, capturing vessel continuity at the cost of adding spurious branches to larger vessels. The **antiga** method provided the highest precision, however, it identified fewer vessels. The method failed to extract skeletons from binarized results using Otsu’s method due to the excessive number of vertices and edges. Gradient-based methods (kerautret and **kline**) performed poorly across most images. While **kline** was designed for vessels, it is poorly suited to microvascular networks since they are not tree-like. The difference in the performance of the kerautret for KESM data also stands out, likely due to the higher resolution of KESM data. This provides (1) a more completely connected mesh and (2) larger distances between adjacent unconnected vessels.

## 7 Discussion

Machine learning (unet, nnunet) achieved the best consistent performance across all images. This is expected given their established performance on semantic segmentation (Wang et al., 2022). While U-Nets have been extensively explored in two-dimensional images, recent advances that mitigate parameter explosion (Saadatifard et al., 2020) make them more practical in 3D. In our experiments, nnU-Net was run in its 3d_fullres mode, which includes isotropic resampling and intensity normalization. Although effective for KESM and µ-CT, this normalization likely suppressed subtle variations unique to LSFM data, reducing the recall and Jaccard index. It is possible that significantly larger training sets could mitigate these issues.

KESM was generally easier to segment due to its high contrast and spatial resolution. In fact, Otsu’s method (**otsu3d**) performed similarly to CNNs on KESM data. KESM also exhibits noise and imaging artifacts that are localized to individual slices, potentially making them easier to ignore. The Vesselness algorithms proposed by Frangi et al. (**frangi**), and later modified by Jerman et al. (**bfrangi**), are the most popular for enhancing tubular structures. While “out-of-the-box” parameters did not perform well, systematic optimization of these parameters (**ofrangi**) provided reasonable performance. However, this requires some form of optimization or training that may not provide a benefit over U-Net. If training data is unavailable, optimally oriented flux (**oof**) provides the best performance.

**FIGURE 14:**
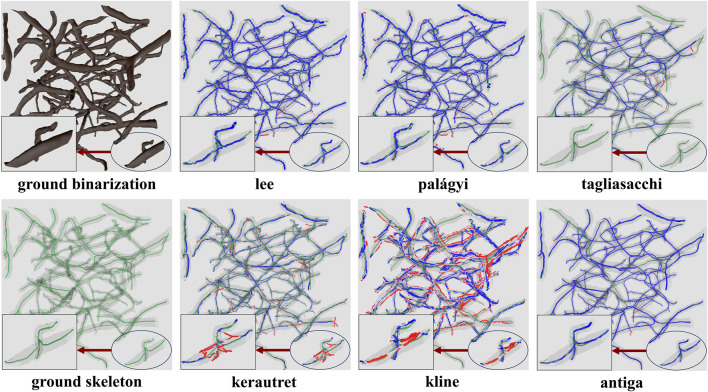
Skeletonization results on the ground truth segmentation of KESM dataset. The ground truth skeleton is colored green in each figure. The skeleton obtained by each method is shown in blue, with false positives marked in red.

For skeletonization (Table 4), both **lee** and palagyi provided consistently good performance. The implementation that we tested for **lee** ^4^ was significantly faster. The **antiga** algorithm provided the best precision for both KESM and µ-CT data. However, this came at a cost to recall since **antiga** tended to miss vessel centerlines (Figure 14). It also performed poorly on LSFM data, likely due to the surface complexity. In fact, the algorithm was unable to skeletonize the LSFM data segmented using **otsu3d**, which exceeded memory limits during the skeletonization process.

**FIGURE 15:**
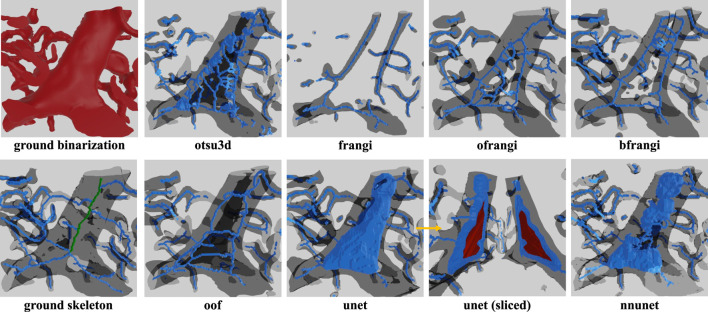
Skeleton extracted using **lee** using different binarizations. One problem frequently encountered in LSFM is hollow vessels that lead to poor recall in the resulting skeletonization. This is due to regions inside large vessels that are misinterpreted as background. This is most commonly encountered in LSFM because the endothelial cells at the vessel surface are common targets for fluorescent labels. This leads to a variety of incorrect “shells” or “networks” that surround the internal gap. The correct medial axis of the hollow vessel is green in the ground truth image.

One of the more interesting findings was that the most accurate binarization (generally a CNN) did not necessarily provide the best input for skeletonization. For example, **otsu3d** µ-CT provided better precision than nnunet when used as an input for LSFM and µ-CT. The reduced precision is likely due to consistent mislabeling of internal vessels (Figure 15). All skeletonization algorithms were also tested on ground truth data (Table 5) to demonstrate potential performance given a highly accurate segmentation.

## 8 Conclusion

This work aims to quantify the performance of algorithms for building large-scale microvascular networks. We focus on data from microscopes with the potential for organ-scale vascular imaging (LSFM, KESM, and µ-CT). We tested two classes of algorithms: binarization and skeletonization. These results can be used to reconstruct microvascular surfaces and study network properties. Importantly, the resulting models can feed directly into post-processing frameworks (e.g., CFD-based flow simulations or perfusion analysis) to facilitate functional interpretation and disease modeling (Celaya-Alcala et al., 2021; Zhang et al., 2022). All of the data, annotations, and links to algorithms are available in a public Git repository.^5^

Although KESM and µ-CT images share high contrast and SNR, their disparate voxel resolutions yield very different results: a global threshold such as otsu performs reliably in KESM, yet degrades in µ-CT where learning-based models like nnunet can incorporate local intensity variations to recover performance. It is important to note that this accuracy is quantified with respect to the ground truth and does not account for the reduced resolution of µ-CT: the accuracy of the segmentation does not necessarily reflect the accuracy of the final model. Modality-specific features, such as pixel sizes, ultimately bound the fidelity of the reconstructed vascular model, even when segmentation metrics appear equivalent across algorithms.

If the user can provide annotated training data, they will likely see the best performance using nnunet (Isensee et al., 2021) for binarization, followed by skeletonization with **lee** (Lee et al., 1994). Users may see an improvement in skeletonization results using **antiga**, provided the vasculature has a lower total surface area.

If a significant amount of training data is unavailable, the authors recommend **oof** (Law and Chung, 2008) for binarization, followed by **lee** for skeletonization. The user may be able to achieve better performance using **frangi** after significant optimization (**ofrangi**) (Frangi et al., 1998).

## Footnotes

1
https://github.com/MIC-DKFZ/nnUNet

2
https://github.com/taiya/starlab-mcfskel

3
https://www.slicer.org/

4 skimage.morphology.skeletonize function in the scikit-image Python library.

5
https://github.com/helia77/MicroVasc-Review
