# Supplementary material for: Segmentation and modeling of large-scale microvascular networks: a survey
Source: Front Bioinform. 2025 Oct 31;5:1645520. doi: 10.3389/fbinf.2025.1645520 (PMC12616183; doi:10.3389/fbinf.2025.1645520)
Supplement: Supplementary file 1 [file DataSheet1.pdf]

frontiers

Supplementary Material

1 SUPPLEMENTARY DATA

Algorithm 1 Vesselness Method in 3D (Frangi et al., 1998)

Load a 3-dimensional image $I(\mathbf{x})$ where $\mathbf{x} \in \mathbb{R}^3$
Specify a scale space sampling rate Δ_σ
Specify the number of scale-space samples S
    scale space components $1 \leq s \leq S$
$\sigma \leftarrow s \cdot \Delta_\sigma$ is the current spatial resolution
$G(\sigma, \mathbf{x})$ is a Gaussian kernel with standard deviation σ dimension pairs $1 \leq i, j \leq 3$
$\mathbf{H}_s(\sigma, \mathbf{x})_{i,j} \leftarrow \sigma^\gamma \frac{\delta^2}{\delta x_i \delta x_j} G(\sigma, \mathbf{x}) \circledast I(\mathbf{x})$ is the Hessian
$\mathbf{Q}\Lambda\mathbf{Q}^{-1} \leftarrow \mathbf{H}_s(\mathbf{x})_{i,j}$ calculate eigendecomposition
$\lambda_i(\mathbf{x}) \leftarrow \Lambda_{ii}$ are sorted such that $\lambda_i \leq \lambda_{i+1}$
$S(\mathbf{x}) \leftarrow \sqrt{\lambda_1^2 + \lambda_2^2 + \lambda_3^2}$ is the Frobenius norm $||\mathbf{H}_s(\mathbf{x})||_F$
$R_A(\mathbf{x}) \leftarrow \frac{|\lambda_2|}{|\lambda_3|}$ detects "plate-like" tensors
$R_B(\mathbf{x}) \leftarrow \frac{|\lambda_1|}{\sqrt{|\lambda_2 \lambda_3|}}$ detects "blob-like" tensors
$L(\mathbf{x}) \leftarrow \left(1 - \exp(-\frac{R_A^2}{2\alpha^2})\right)$ response to "lines"
$B(\mathbf{x}) \leftarrow \exp\left(-\frac{R_B^2}{2\beta^2}\right)$ inverse response to "blobs"
$K(\mathbf{x}) \leftarrow \left(1 - \exp(-\frac{S^2}{2c^2})\right)$ suppresses background
$V_s(\mathbf{x}) \leftarrow 0$ light vessels on dark background
$V_s(\mathbf{x}) \leftarrow L(\mathbf{x})B(\mathbf{x})K(\mathbf{x})$ if $\lambda_2, \lambda_3 \leq 0$
$V_s(\mathbf{x}) \leftarrow L(\mathbf{x})B(\mathbf{x})K(\mathbf{x})$ if $\lambda_2, \lambda_3 \geq 0$
$V(\mathbf{x}) = \max_{s \in [1,S]} V_s(\mathbf{x})$

Algorithm 2 Beyond Frangi - (Jerman et al., 2016)

$\rightarrow$ **Execute** Vesselness filter (Algorithm 1)

scales s

$\bar{\lambda}(s) \leftarrow \max_{\mathbf{x}} [|\lambda_3(\mathbf{x}, s)|]$ is the largest λ_3 pixels $\mathbf{x}$ and scales s

Re-scale all λ_3 eigenvalues: $|\lambda_3(\mathbf{x})| > \tau|\bar{\lambda}(s)|$

$\lambda_\rho(s, \mathbf{x}) \leftarrow \lambda_3(\mathbf{x})$ $0 < |\lambda_3(\mathbf{x})| \leq \tau|\bar{\lambda}(s)|$

$\lambda_\rho(s, \mathbf{x}) \leftarrow \tau\bar{\lambda}(s)$

$\lambda_\rho(s, \mathbf{x}) \leftarrow 0$

$\mathbf{V}_J(s, \mathbf{x}) \leftarrow \lambda_2^2(\lambda_\rho - \lambda_2)\left[\frac{3}{\lambda_2+\lambda_\rho}\right]^3$ "beyond Frangi" filter

$0 < |\lambda_\rho|/2 \leq |\lambda_2|$

$\mathbf{V}_J(s, \mathbf{x}) \leftarrow 1$ light vessel on dark background

$V_J(s, \mathbf{x}) \leftarrow 0$ if $\lambda_2, \lambda_3 \geq 0$ dark vessel on light background

$V_J(s, \mathbf{x}) \leftarrow 0$ if $\lambda_2, \lambda_3 \leq 0$

$V(\mathbf{x}) = \max_{s\in[1,S]} \mathbf{V}_J(s, x)$

Algorithm 3 Lee's method (Lee et al., 1994)

Load a 3-dimensional binarized image $I = \{V, \hat{V}\}$

V consists voxels with value 1, $\hat{V}$ is the complement voxel $v = (i, j, k)$ in image I

$N(6) \leftarrow$ direct 6-adjacent points

$N_i^2(v) \leftarrow$ "octant" in $N(26)$ where $1 \leq i \leq 8$ $v \in V$ and $0 \in N(6)$

check if it is Euler invariant.

check if not an end-point $\rightarrow \sum_{i=1}^{8} N^2(v) \neq 1$

check if it is simple point by $N(v)$ labeling method

N(v) labeling algorithm: p in $N(26)$ $p = 1$

run Octree labeling

output: number of connected object in $N(26)$

Octree labeling:

$p' = 1$ in current octant

$p' \leftarrow$ label value

run *Octree labeling* for adjacent octant

output: labeled volume

not a *simple point* if number of connected object ≥ 1

REFERENCES

Frangi, A. F., Niessen, W. J., Vincken, K. L., and Viergever, M. A. (1998). enMultiscale vessel enhancement filtering. In *Medical Image Computing and Computer-Assisted Intervention — MICCAI'98*, eds. W. M. Wells, A. Colchester, and S. Delp (Berlin, Heidelberg: Springer), Lecture Notes in Computer Science, 130–137. doi:10.1007/BFb0056195

Jerman, T., Pernuš, F., Likar, B., and Špiclin, Ž. (2016). Enhancement of vascular structures in 3d and 2d angiographic images. *IEEE Transactions on Medical Imaging* 35, 2107–2118. doi:10.1109/TMI.2016.2550102

Lee, T. C., Kashyap, R. L., and Chu, C. N. (1994). enBuilding Skeleton Models via 3-D Medial Surface Axis Thinning Algorithms. *CVGIP: Graphical Models and Image Processing* 56, 462–478. doi:10.1006/cgip.1994.1042
